# The Asylum World

**Published:** 1910-09-24

**Authors:** 


### THE ASYLUM WORLD.

### REPORTS OF PROGRESS DURING THE PAST YEAR.

#### Broadmoor Criminal Lunatic Asylum.

During the year 843 patients, 611 males and 232 females, were under treatment at this asylum. New cases admitted amounted to 42, of which eight were females. The crimes and offences of these patients were as follows: Murder, 23 (seven females being among this number); attempted murder, maim, etc., ten; unnatural offences, one; indecent assault on females, one; burglary, two; larceny, three (one female among these); and arson, two. Of these patients four showed an insane heredity, eight congenital mental instability, three were afflicted with mental disturbance following the puerperium and lactation, one developed symptoms as the result of sudden and of prolonged mental stress. Alcohol accounted for the admission of nine; injury or sunstroke for that of three others. Epilepsy led to the incarceration of three cases, another suffered from a brain lesion, and in nine cases the past history was doubtful. The death-rate per cent. worked out at 3.01, there being twenty-four deaths (males twenty-two, females two); the average age at death was 67.75 for males and 67 for females. The causes of death were as follows:—Pneumonia, one; sarcoma, one; carcinoma, one; senile decay, six; cerebral congestion, one; heart disease, three; cerebral hæmorrhage, one; icterus, one; chronic Bright's disease, three; and strangulated hernia, one. There were sixteen cases discharged during the year, of whom nine were females. With the exception of one male, who on recovery was sent back to prison, the remainder discharged were given their liberty. Among patients still under control in the asylum, are two suffering from general paralysis, thirty-seven from epilepsy, and seven from paralysis. The medical staff of the institution consists of a superintendent, under whom work three assistant medical officers, 107 male attendants, and thirty-six nurses, in addition to a large administrative and clerical personnel. The cost per head for the maintenance of patients works out at an average of £39 10s. 9d. per annum. The report of the Commissioners in Lunacy with regard to this asylum draws attention to overcrowding among the female patients and bad ventilation in some of the buildings. On January 1, 1909, there were under treatment 801 patients (577 males and 224 females), and on December 31 the number was the same (581 males and 220 females).

#### West Riding Pauper Lunatic Asylum, Wakefield.

First opened for the reception of patients in 1818, this asylum now has accommodation for 2,023 inmates. Medical treatment is in the hands of a superintendent, with a staff of six assistant medical officers under him. The nursing staff is sufficient to provide during the daytime one attendant or nurse for every 9.5 patients. There came under medical treatment in 1909, 2,496 patients, of whom 289 males and 237 females were new cases. The mental disorders for which these new patients were admitted comprised the following: Fifty-three were congenital cases, of which seventeen were cases of intellectual insanity with epilepsy; thirty-three were intellectually insane, but without epilepsy; and three were morally insane. Of the remaining 473 cases in which the mental disease was acquired 57 suffered from general paralysis, 35 from insanity with epilepsy, 6 from acute delirium, 11 from confusional insanity, 17 from stupor, 25 from primary dementia, 94 from mania (recent 83, chronic 6, recurrent 5), 86 from melancholia (recent 72, chronic 12, recurrent 2), 6 from alternating insanity, 63 from delusional insanity, 4 from volitional insanity, 4 from moral sanity and 63 from dementia (senile 46, secondary 17). Of those under treatment during the year 58 patients were 15 years of age or under, and 210 were 65 or over. Deaths at the institution accounted for 116 males and 79 females, giving a death-rate of 7.81 per cent. of those under treatment. The principal causes of death, among others, were general paralysis 49, phthisis 37, heart disease 27, cardio-vascular degeneration 23, brain atrophy 22, nephritis 17, pneumonia 14, etc. Recovery accounted for the discharge of 208 patients, 107 males and 101 females. In addition 31 males and 70 females were discharged without showing improvement. The percentage of recoveries to admissions was 37.02 in males and 46.62 in females. The asylum has a flourishing pathological department under the care of Dr. G. S. Williamson, who has carried out important work there with regard to the pathology of insanity. Good results have been obtained in the electro-therapeutic department. Treatment by means of the sinusoidal current resulted in recovery in 143 cases, relief in 40, and no improvement in 69. High-frequency currents and static baths accounted for cures in 48, relief in 28, and no improvement in 17 cases. The weekly cost per head for maintenance of the patients amounted to 10s. 1¼d. The report of the Commissioners in Lunacy was in every way to the credit of the institution. On January 1, 1909, there were 1,970 patients (1,096 males, 874 females) in the asylum. On December 31 the number was 1,992 (1,131 males, 861 females).

County and City of Worcester Lunatic Asylum, Powick.

During the year under review 1,069 patients received treatment, of whom 481 were males and 582 females. Of the 109 new cases admitted to the asylum 61 were males, 48 females. Congenital mental deficiency accounted for 10 of the new cases, all being of the intellectual type without epilepsy. Of the remaining 99 cases, 2 were due to insanity with epilepsy, 3 to general paralysis of the insane, 60 to mania (42 recent, 18 chronic), 25 to melancholia (24 recent, 1 chronic), 1 to volitional impulsive insanity, and 8 to dementia (5 senile, 3 secondary). Seven males and 14 females had already had a previous attack of insanity. During the year 77 deaths occurred (37 males and 40 females), giving a death-rate per cent. of those under treatment of 8.2. Causes of death were the following : Phthisis 10, pneumonia 9, general paralysis 8, cancer of stomach 1, intestinal tuberculosis 1, cancer of liver 2, cancer of uterus 1, facial erysipelas 2, congenital hydrocephalus 1, exhaustion from mania 1, cerebral abscess 1, epilepsy 2, cerebral softening 2, heart disease 29, vascular disease 5, respiratory disease 5, peritonitis 2, splenic anæmia 1, senile decay 13, asphyxia by suffocation 1. Discharges and transfers accounted for 53 cases. Of these 34 recovered (males 14, females 20), 4 were relieved (1 male). The staff of the asylum consists of a medical superintendent, 3 assistant medical officers, 94 day and 16 night attendants and nurses, besides the clerical and administrative staff. The weekly cost per head for maintenance of the patients averaged 9s. 1½d. net, and 10s. 8d. gross (including interest on loans, repayment of capital, etc.). In their report the Commissioners in Lunacy draw attention to the need there is that repairs should be done to certain floors, as well as that carpets and linoleums and paint in many places should be renewed. They also point out the undesirability of treating phthisical patients in the general dormitories, and suggest that special accommodation be provided in future for such cases. On January 1, 1909, 950 cases were under treatment in the asylum, comprising 420 males and 534 females. On December 31 the number was 933 (425 males and 508 females).

James Murray's Royal Asylum, Perth.

This asylum is an endowed institution and a chartered corporation under the management of a statutory board of directors, who have no pecuniary interest in its prosperity. Such profits as are made go to improvements in the fabric and the provision of new buildings as required. In addition to the main building, but quite separated from it, is a convalescent home for patients recovering, as well as for mild cases. The institution receives no pauper patients. Medical treatment is in the hands of a physician superintendent, who has associated with him an assistant medical officer, twenty-two male attendants, and twenty-four nurses, in addition to the usual administrative staff. During the year 179 patients received treatment, there being 40 new cases, of whom 14 were males. Congenital mental deficiency accounted for two of these admissions, melancholia for 14 (12 recent, two chronic), circular insanity for one, delusional insanity for two, confusional insanity for one, and dementia for six. Fourteen deaths occurred during the year, which gave a death-rate among the inmates of 10.25 per cent. The causes of death were as follows : General paralysis 2, exhaustion from mania 1, influenzal pneumonia 4, chronic heart disease 3, kidney disease 1, disease of gall bladder 1, tuberculous peritonitis 1, burns received prior to admission 1. Thirty persons were discharged, of whom 12 were men. Thirteen patients left the institution restored to health. The report on the Asylum of the Commissioners in Lunacy was in every way satisfactory. On January 1, 1909, 127 persons were under treatment in the institution, of whom 63 were males and 64 females. On December 31 the number was 130, of whom 64 were males and 66 females.

Glamorgan County Lunatic Asylum, Bridgend.

This asylum has two branches, one at Angelton, the other at Parc Gwyllt. Treatment is in the hands of a medical superintendent, under whom are four assistant medical officers, and a staff of attendants and nurses sufficient to provide one of either for every 9.8 patients. During the year 2,101 patients were under treatment, 1,182 being males, and 910 females. New cases amounted to 364, of whom 199 were males. Of new admissions 33 were congenital, 10 being afflicted with intellectual insanity with epilepsy, 22 with the same without epilepsy, there being one case of congenital moral insanity. In the acquired cases 23 suffered from insanity with epilepsy, 27 from general paralysis, 10 from insanity with gross brain lesions, one from acute delirium, six from confusional insanity, 148 from mania (recent 116, chronic 32), 91 from melancholia (recent 73, chronic 18), eight from delusional insanity, one from moral insanity, and 15 from dementia (senile 10, secondary 5). The death-rate worked out at an average of 10.6 per cent., there being 176 deaths. The principal causes of death were : General paralysis 28, tuberculosis of lungs 24, senile decay 22, heart disease 19, chronic nephritis 14, pneumonia 13, etc. There were discharged 129 cases, of whom 96 were described as cured, 31 relieved, and two not improved. There were 158 transfers, of whom 24 were relieved and 134 not benefited. One male patient succeeded in committing suicide by cutting his throat, and another patient, a female melancholic, attempted the same, but was not successful. The average weekly cost per head for maintenance of the patients worked out at 10s. 0⅝d. The report of the Commissioners in Lunacy was satisfactory to those concerned in the administration of the asylum. On January 1, 1909, there were 1,737 patients (983 male and 754 female) in the institution, and on December 31 there were 1,638 (870 males and 768 females).

Roxburgh, Berwick, and Selkirk District Board of Lunacy.

Situated at Melrose, the asylum administered by this board dealt with 376 patients during the 12 months under review. Of these 52 were new cases (30 males and 22 females). One case of congenital mental deficiency without epilepsy was admitted. The remaining cases of acquired insanity included insanity with epilepsy 1, general paralysis 2, insanity with gross cerebral lesions 2, mania 16 (recent 11, chronic 4, recurrent 1), melancholia 20 (recent 17, chronic 3), delusional insanity 4, dementia 6 (senile 2, secondary 4). Deaths amounted to 23, cerebral hæmorrhage accounting for 2, epilepsy 1, general paralysis 2, softening of the brain 5, heart disease 1, pulmonary tuberculosis 9, pyonephrosis 1, Bright's disease 1, cancer of stomach 1. During the twelve months the death rate worked out at 7.1 per cent. of those under treatment. Thirty-four patients were discharged, of whom 23 (12 males and 11 females) had recovered and 11 (6 males and 5 females) were relieved, giving a proportion of recoveries to admissions of 44.2 per cent. The weekly cost of maintenance per head of patients under treatment averaged £1 3s. 8$\frac{1}{16}$d. The report of the Commissioners in Lunacy on the asylum was satisfactory to those concerned in its administration. There were under treatment on May 15, 1909, in the institution 299 patients, of whom 151 were males and 148 females. On May 15, 1910, the total number in residence was 295, males being 153, females 142.

